# Gamification for Distal Radius Fracture Rehabilitation: A Randomized Controlled Pilot Study

**DOI:** 10.7759/cureus.29333

**Published:** 2022-09-19

**Authors:** Waqar M Naqvi, Moh'd Irshad Qureshi, Gargi Nimbulkar, Laxmikant Umate

**Affiliations:** 1 Physiotherapy, Ravi Nair Physiotherapy College, Datta Meghe Institute of Medical Sciences, Wardha, IND; 2 Directorate of Research, N.K.P. Salve Institute of Medical Sciences and Research Center, Nagpur, IND; 3 Neuro-Physiotherapy, Ravi Nair Physiotherapy College, Datta Meghe Institute of Medical Sciences, Wardha, IND; 4 Department of Preventive and Community Dentistry, Raj Rajeshwari Dental College and Hospital, Udaipur, IND; 5 Department of Research and Development, Datta Meghe Institute of Medical Sciences, Wardha, IND

**Keywords:** functional independence, grip strength, pain, pilot study, virtual reality, physiotherapy, distal radial fracture, oculus quest, gamification

## Abstract

Introduction

Gamification is a novel interventional approach to functional recovery and rehabilitation. A significant impact has been observed with the application of gamification on non-traumatic conditions and chronic neurological and musculoskeletal illnesses; however, the implication of gamification on the functional recovery of patients with distal radius fractures (DRF) is yet to be explored.

Methodology

This pilot study included 20 post-DRF patients aged 18-65 years with unilateral DRF, managed with closed reduction and K-wire internal fixation. The patients were assigned to group A (gamification) and group B (conventional rehabilitation) in a 1:1 ratio. Group A patients played Racket: NX game, Until you fall game, and Holofit game on Oculus Quest head-mounted display (HMD) (Oculus, USA), while group B patients received a conventional rehabilitation program. Both groups underwent a rehabilitation program for 60 min/day, five days a week, for four weeks. The visual analogue scale (VAS), universal goniometer, Jamar dynamometer, and Disabilities of the Arm, Shoulder, and Hand (DASH) questionnaire were used as outcome measures at baseline, at the end of the second week, and at the end of treatment.

Results

There were significant improvements in pain, range of motion (ROM), grip strength, and functional independence in both groups. However, improvements in hand function and functional independence were significantly greater in the gamification group than in the conventional physiotherapy rehabilitation group.

Conclusion

The study concluded that gamification appears to have a significant impact on post-DRF rehabilitation in terms of pain, ROM, grip strength, and functional independence. Further research with larger sample sizes is required to confirm the preliminary findings.

## Introduction

Gamification is the implication of serious gaming in functional rehabilitation as a novel intervention technique [1]. It has been shown to have a positive effect in patients with chronic lower back pain [2], lower limb amputation [3], traumatic shoulder stiffness [4], cerebral palsy [5], traumatic brain injury [6], and other musculoskeletal disorders [7]. The efficacy of gamification was applied and analyzed on a patient with distal radius fracture (DRF) and was found to be effective in reducing pain, improving range of motion (ROM), the strength of grip, and functional independence [8]. Therefore, its efficacy has been explored for determining the feasibility of its application in the rehabilitation of DRF.

Post-DRF patients usually experience pain, restricted ROM, reduced grip strength, and limited functional independence due to immobilization under a plaster cast for four-six weeks immediately after the fracture [9]. Therefore, the study aimed to compare the impact of gamification in post-DRF rehabilitation. 

## Materials and methods

This feasibility pilot study was conducted in the Department of Physiotherapy, Acharya Vinoba Bhave Rural Hospital (AVBRH), Datta Meghe Institute of Medical Sciences University (DMIMSU), Wardha, Maharashtra, India. The Institutional Ethical Committee (IEC) approved this research with Ref.No. DMIMS(DU)/IEC/2022/996. The conduction of the study followed the Declaration of Helsinki. The study protocol has been deposited in the repository (http://hdl.handle.net/123456789/9990) [10].

Post-DRF patients referred from the Department of Orthopaedics were recruited in this pilot trial. The DRF aligned with open reduction and internal fixation (ORIF) surgically using external fixation and volar plate, and patients with a Mini-Mental score less than 26 points on the examination were excluded. Patients with immediate complications such as malunion or non-union, impaired hand function following any past trauma to the hands or arms, inflammatory or non-inflammatory diseases, or neurological disorders were also excluded.

After screening for eligibility criteria and obtaining informed consent, 20 post-DRF patients aged 18-65 years with unilateral DRF, managed with closed reduction and K-wire internal fixation were recruited. The patients were randomized using computer-generated randomization and assigned to group A (gamification) and group B (conventional rehabilitation) in a 1:1 ratio using a sequentially numbered, sealed opaque envelope method. Group A patients played 'Racket: NX' game, 'Until you fall' game, and 'Holofit' game on Oculus Quest head-mounted display (HMD) (Oculus, USA), while group B patients received a conventional rehabilitation program. Both groups underwent a therapeutic guided rehabilitation for sixty minutes per day five days per week for four weeks. The visual analog scale (VAS), universal goniometer, Jamar dynamometer, and Disabilities of the Arm, Shoulder, and Hand (DASH) questionnaire were used as outcome measures and were measured at baseline (t0), the end of the second week (t2) and at the end of the treatment (t4) [8]. The scores obtained at t0, t2, and t4 were measured and compared within and between the groups.

## Results

In group A, 20% of patients belonged to the age group 18-25 years, 10% belonged to 26-35 years, 50% belonged to 36-45 years, and 20% belonged to 56-65 years with 80% males and 20% females. In group B, 30% belonged to the age group 18-25 years, 10% belonged to 26-35 years, 30% belonged to 36-45 years, and 30% belonged to 56-65 years with a 50% male-female distribution. The gender-wise distribution between the groups was statistically analyzed using the Chi-square test and found to be insignificant (p=0.16) with χ2=1.978.

The pain intensity was assessed using VAS and compared at t0, t2, and t4. This was statistically analyzed using the repeated measure analysis of variance (ANOVA) test and demonstrated a significant difference (p<0.001) statistically in the intensity of pain at t0, t2, and t4. The improvement between the groups was statistically analyzed with an unpaired t-test and found to be statistically insignificant at t0 (p=0.08) but significant at t2 and t4 (p<0.001) (refer to Table 1). 

**Table 1:** Comparative evaluation of mean Visual Analog Scale score between two groups

Groups	VAS score (Mean ± SD)			p-value intragroup		
	Baseline	2^nd^ week	4^th^ week	Baseline-2^nd^ week	Baseline-4^th^ week	2^nd^ week-4^th^ week
Group A	6.99±0.74	3.74±0.72	1.77±0.38	<0.001**	<0.001**	<0.001**
Group B	7.52±0.50	5.98±0.39	4.18±0.27	<0.001**	<0.001**	<0.001**
p-value (intergroup)	0.08 NS	<0.001**	<0.001**	-	-	-

**Figure 1:**
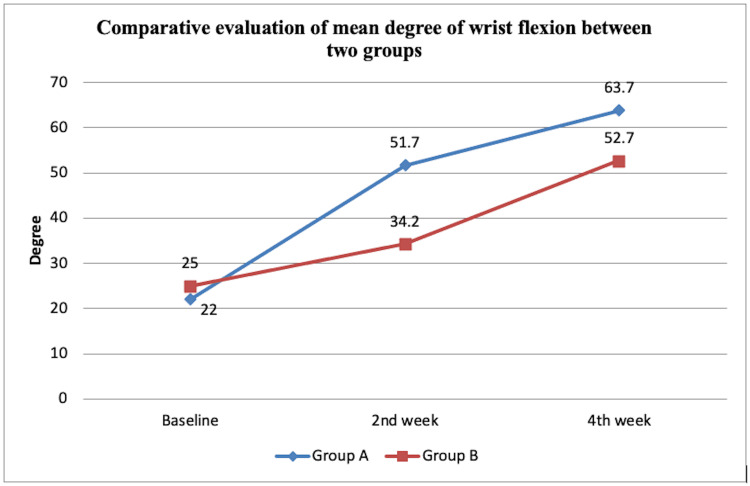
Comparative evaluation of the mean degree of wrist flexion between two groups

The wrist ROM was assessed using a Universal goniometer. The intragroup mean and standard deviation (SD) were compared at t0, t2, and t4 as 22.00 ±8.56, 51.70 ±7.34, and 63.70 ±3.88 respectively for group A and for group B, the mean difference with SD was recorded as 25.00 ±8.16 at t0, 34.20 ±5.37 at t2, and 52.70 ±3.91 at t4. The intragroup mean difference was compared and statistically analyzed using the ANOVA test and the intergroup findings were compared and statistically analyzed using an unpaired t-test. Both groups A and B demonstrated significant differences (p<0.001) statistically in intragroup and intergroup analysis for wrist flexion. Figure 1 illustrates the comparative evaluation of the mean degree of wrist flexion between the two groups. 

Following wrist flexion, statistical analysis of wrist extension ROM at t0, t2, and t4 was done using the ANOVA test and obtained as significant (p<0.001) statistically. The unpaired t-test was used for statistical analysis at the intergroup level showing a significant difference (p<0.001) statistically except at baseline (p=0.551) (Table 2).

**Table 2:** Comparative evaluation of the mean degree of wrist extension between two groups

Groups	Degree of Wrist Extension (Mean ± SD)			p-value intragroup		
	Baseline	2^nd^ week	4^th^ week	Baseline-2^nd^ week	Baseline-4^th^ week	2^nd^ week-4^th^ week
Group A	13.30 ±4.24	43.60 ±5.75	63.70 ±3.88	<0.001**	<0.001**	<0.001**
Group B	14.50 ±4.57	34.80 ±3.11	52.70 ±3.91	<0.001**	<0.001**	<0.001**
p-value (intergroup)	0.551 NS	<0.001**	<0.001**	-	-	-

**Figure 2:**
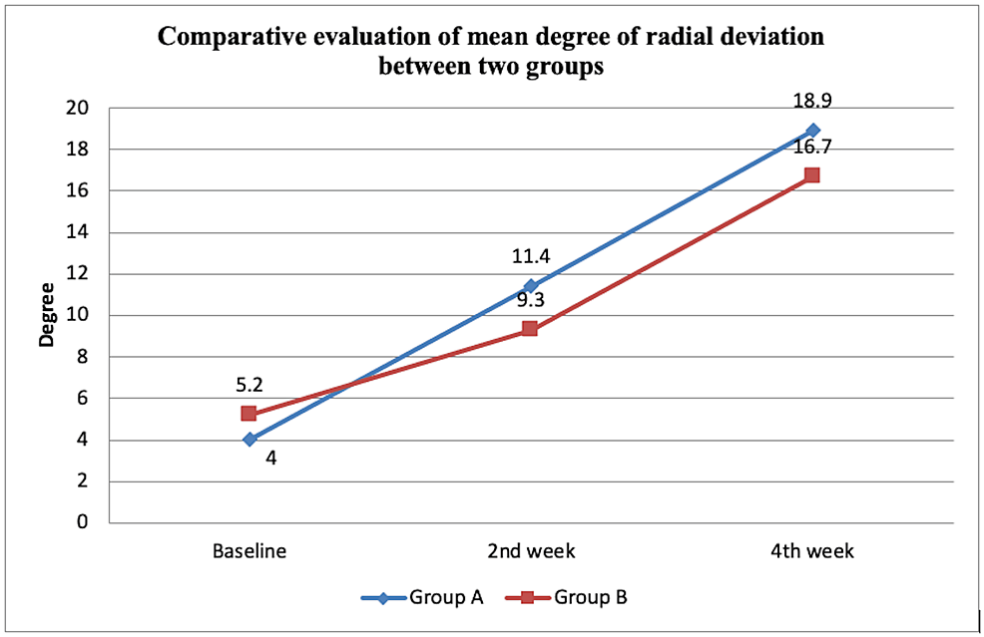
Comparative evaluation of the mean degree of radial deviation between two groups

Similarly, the mean difference with SD of radial deviation for group A at t0, t2, and t4 was 4.00 ±4.61, 11.40 ±2.45, and 18.90 ±1.66 respectively. The mean difference with SD for group B was 5.20 ±4.02 at t0, 9.30 ±2.11 at t2, and 16.70 ±2.00 at t4. The ANOVA test was used to statistically analyze the intragroup findings suggesting a statistically significant (p<0.001) difference. The intergroup statistical analysis was done using an unpaired t-test and was also found to be statistically significant (p<0.001) except for baseline (p=0.543) and at t2 (p=0.055). Figure 2 demonstrates the comparative evaluation of the mean degree of wrist radial deviation between the two groups.

The statistical analysis of ulnar deviation ROM at t0, t2, and t4 was done using the ANOVA test which demonstrated a statistically significant (p<0.001) difference. The unpaired t-test analyzed intergroup findings showing statistically significant (p<0.001) difference except for baseline (p=0.419) and at t2 (p=0.007) (Table 3). 

**Table 3:** Comparative evaluation of the mean degree of ulnar deviation between two groups

Groups	Degree of Ulnar Deviation (Mean ± SD)			p-value intragroup		
	Baseline	2^nd^ week	4^th^ week	Baseline- 2^nd^ week	Baseline- 4^th^ week	2^nd^ week-4^th^ week
Group A	11.90 ±2.68	29.30 ±2.35	44.80 ±3.39	<0.001**	<0.001**	<0.001**
Group B	10.90 ±2.72	24.90 ±3.87	35.70 ±3.86	<0.001**	<0.001**	<0.001**
p-value (intergroup)	0.419 NS	0.007*	<0.001**	-	-	-

**Figure 3:**
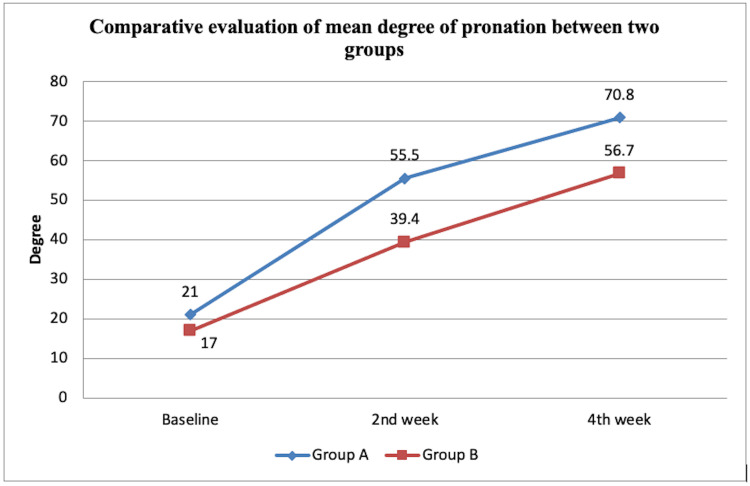
Analysis of mean range on comparison of pronation in between groups A and B

The mean degree with SD of pronation for group A was found to be 21.00±7.74 at t0, 55.50±4.45 at t2, and 70.80±5.22 at t4 however for group B, the mean degree with standard deviation was 17.00±8.56 at t0, 39.40 ±0.96 at t2, and 56.70 ±3.05 at t4. The statistical analysis of pronation at t0, t2, and t4 was done using the ANOVA test which demonstrated a statistically significant (p<0.001) difference. The unpaired t-test was used for intergroup statistical analysis stating a statistically significant (p<0.000) difference except for baseline (p=0.288). Figure 3 demonstrates the analysis of the mean range on comparison of pronation in groups A and B.

Similarly, the statistical analysis of supination at t0, t2, and t4 was done using the ANOVA test which demonstrated a statistically significant (p<0.001) difference. The unpaired t-test was used for intergroup statistical analysis stating a significant (p<0.000) difference statistically except for baseline (p=0.775) (refer to Table 4). 

**Table 4:** Analysis of mean range on comparison of supination in between groups A and B

Groups	Degree of Supination (Mean ± SD)			p-value intragroup		
	Baseline	2^nd^ week	4^th^ week	Baseline-2^nd^ week	Baseline-4^th^ week	2^nd^ week-4^th^ week
Group A	23.50 ±7.83	44.20 ±3.79	68.70 ±4.57	<0.001**	<0.001**	<0.001**
Group B	22.50 ±7.54	33.80 ±3.55	59.40 ±1.83	<0.001**	<0.001**	<0.001**
p-value (intergroup)	0.775	<0.001**	<0.001**	-	-	-

The comparison of the DASH score was done to assess the functional independence at t0, t2, and t4 and was statistically analyzed using the ANOVA test. The mean DASH score was statistically significant (p<0.001) at the intragroup level. The intergroup mean with SD was analyzed using an unpaired t-test as statistically significant (p<0.001) except at baseline (p=0.417) (refer to Table 5). 

**Table 5:** Comparative analysis of mean DASH score between groups A and B DASH: Disabilities of the Arm, Shoulder, and Hand

Groups	DASH score (Mean ± SD)			p-value intragroup		
	Baseline	2^nd^ week	4^th^ week	Baseline-2^nd^ week	Baseline-4^th^ week	2^nd^ week-4^th^ week
Group A	80.16 ±1.65	37.58 ±2.81	12.95 ±1.72	<0.001**	<0.001**	<0.001**
Group B	80.68 ±2.81	49.24 ±1.93	28.10 ±6.57	<0.001**	<0.001**	<0.001**
p-value (intergroup)	0.417 NS	<0.001**	<0.001**	-	-	-

**Figure 4:**
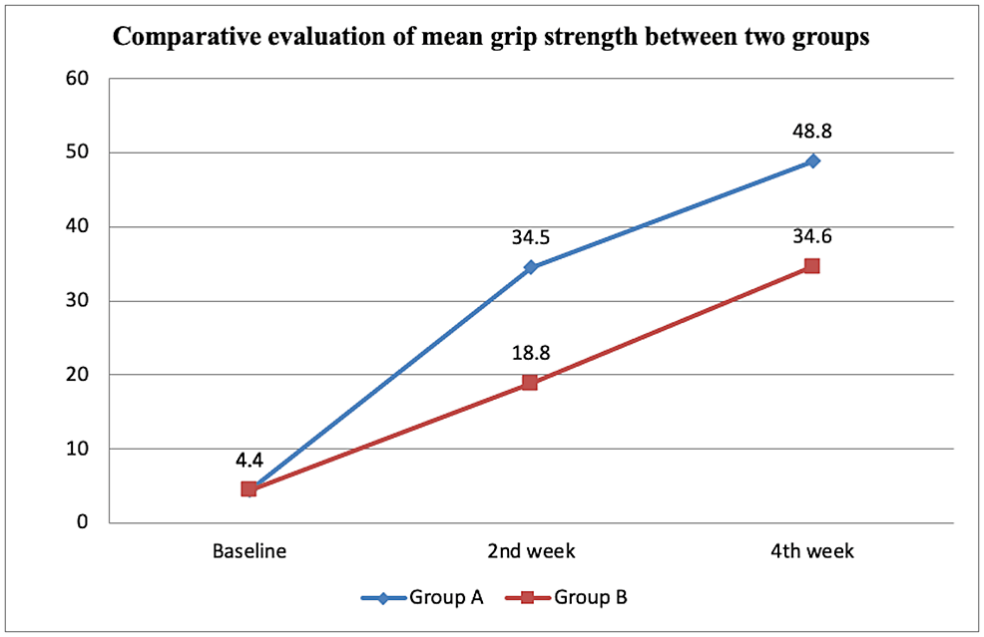
Comparative analysis of mean grip strength between groups A and B

The mean grip strength with SD was evaluated at t0 as 4.40 ±2.06, t2 as 34.50 ±3.74, and t4 as 48.80 ±4.34 for group A. For group B, it was evaluated at t0 as 4.40 ±2.06, t2 as 18.80 ±1.68, and t4 as 34.60 ±3.53. The intragroup findings were compared and analyzed using the ANOVA test to be significant (p<0.001) statistically. The intergroup comparison was statistically analyzed using an unpaired t-test as statistically significant (p<0.001) except at baseline (p=1.0). Figure 4 illustrates the comparative analysis of mean grip strength in groups A and B.

Statistical analysis was conducted by descriptive and inferential statistics using the Chi-square test, Student’s unpaired t-test, and repeated measure ANOVA test. Data analysis was done using IBM Statistical Package for Social Sciences, Statistics for Windows, Version 21.0. (IBM Corp., Armonk, NY) and GraphPad Prism 7.0 version (GraphPad Software, San Diego, CA), and p<0.05 was considered as a level of significance.

## Discussion

A study published earlier has been followed to derive the interventional regimen followed in this trial [8] and analyzed for the feasibility of a limited sample size by plotting the piloting for a larger sample size. The confounding factors showed the distribution of DRF incidence and encounter as per inclusion criteria in different age groups with a statistically insignificant gender-wise distribution. Similar findings were noted previously by MacIntyre and Dewan in 2016 while stating the stratification of risk factors, epidemiology, and prognosis of DRF [11]. 

This study found that both interventional methods have an effective impact on pain but highlighted the enhanced effect of gamification in reducing the intensity of pain post DRF. In 2016, Jones et al. reported an effective relief in pain using an HMD-induced virtual reality (VR) experience on chronic pain [12], and in 2007, Gold et al. described the mechanism underlying in attenuation of pain at the neurobiological level relieving acute pain [13]. 

The improvement in ROM is reported in both groups however group A demonstrated a comparatively higher degree of freedom supporting the findings of Then et al. who reported composite significance in improving finger ROM by using gamification induced with a mobile device post metacarpal fracture [14]. In 2022, Chen et al. reported that VR-supported therapy had a significant improvement in ROM [15]. 

Similarly, functional independence illustrated a significant improvement in the DASH score supported by the study done in 2020 by Pazzaglia et al. stating that VR-based rehabilitation is more effective in improving functional independence as compared to conventional programs [16]. Also, in 2022, Schwartz et al. conducted a study determining the manipulation-enabled virtual kinematic intervention for traumatic shoulder stiffness and concluded that manipulated virtual intervention is more beneficial in improving functional independence [4]. However, in 2017, Laver et al. in a systematic review stated that VR could be useful in improving the functional independence of upper extremities solely when used in conjunction with standard rehabilitation treatment [17].

The grip strength also showed a statistically significant improvement in both the groups with a more significant mean difference in the gamification group. A study in 2016 by Lee et al. assessed the efficacy of VR training on bilateral upper extremity and concluded an impactful improvement in grip strength along with palmar, tip, and lateral pinch [18]. However, in 2020, Then et al. conducted a study comparing conventional therapy with gamification and found no significant grip strength improvement [14].

Apart from the variables of evidence, gamification provides an engaging, interesting, interactive, and easy-to-execute platform for motor and cognitive rehabilitation [19]. The real-time feedback distracts a limited degree of freedom and provides an immersive environment for achieving the restricted hand function [20]. Gamification has a wide spectrum of applications in different conditions with different rehabilitation aims, however, its implication is to be validated on a larger sample size in further research. The heterogeneous distribution of participants, intervention, outcomes, and implications put forth the limitation of the study. The outcomes can be explored and followed-up on a long-term basis stating the future implication with exclusive effectiveness of individual games.

## Conclusions

The study concludes that gamification appears to have a significant impact on post-DRF rehabilitation in terms of relief in pain, improved ROM, strength of grip, and functional independence. The initial results are indicating that gamification can be immersive, interesting as well as an efficient treatment method for the patients with upper limb patients. The finding of this pilot study suggests that the impact of gamification on post-DRF patients can be studied in larger and more diverse populations.
